# Case report: A case report and literature review on the efficacy of high-dose aumolertinib combined intrathecal pemetrexed by Ommaya reservoir for EGFR-mutated NSCLC with leptomeningeal metastasis as the initial symptoms

**DOI:** 10.3389/fonc.2025.1502934

**Published:** 2025-01-30

**Authors:** Maoxi Zhong, Li Zhou, Jing Guo, Chuan Chen, Yi Liu, Xiaoping Huang, Wei Wang

**Affiliations:** 1 Department of Cancer Center, Chongqing University Three Gorges Hospital, Chongqing, China; 2 School of Medicine, Chongqing University, Chongqing, China; 3 Chongqing Municipality Clinical Research Center for Geriatric Diseases, Chongqing, China; 4 Department of Thoracic Surgery, Chongqing University Three Gorges Hospital, Chongqing, China

**Keywords:** high-dose aumolertinib, intrathecal chemotherapy, leptomeningeal metastasis, NSCLC, case report

## Abstract

Leptomeningeal metastasis (LM) is a significant complication of advanced non-small cell lung cancer (NSCLC), occurring in only 3-5% of patients and exceedingly rare in newly diagnosed NSCLC patients. This also indicates that the tumor is highly malignant and aggressive, which brings great challenges to treatment. Here we present a case report of an EGFR-mutated NSCLC patient who presented with LM as the primary clinical manifestation, and review the latest advances in existing studies on LM-related treatment. The patient underwent multiple cycles of high-dose aumolertinib in combination with intrathecal pemetrexed administered via Ommaya reservoir. As of the submission date, the patient achieved significant remission and a LM Progression-Free Survival (PFS) exceeding 20 months. This case highlights the positive impact of high-dose aumolertinib combined with intrathecal pemetrexed on NSCLC patients presenting with severe meningeal symptoms as the initial manifestation, offering a viable therapeutic approach for managing severe meningeal symptoms associated with LM, such as headache, nausea, neck stiffness, and vomiting.

## 1 Introduction

LMoccurs when tumor cells spread into the leptomeninges, subarachnoid space, and other cerebrospinal fluid (CSF) chambers. NSCLC is one of the most common solid tumors with LM. Clinical manifestations are diverse and non-specific, primarily including neurological symptoms in three aspects, increased intracranial pressure (dizziness, headache, vomiting, and optic neuroid edema), meninges irritation (cervical stiffness, Kernig’s sign, and Brudzinski’s sign), and cerebral neuropathy (1). The diagnosis of LM is based on clinical findings, head MRI, and CSF cytology. Once LM develops, the patient may rapidly progress toward death. Modern treatments have increased the OS from 1-3 months to 3-11 months (1, 2), but treatment remains extremely challenging. LM patients are heterogeneous due to complex etiology and molecular characteristics. The optimal approach remains uncertain, particularly for those with driver gene mutations, leaving many questions about precise and personalized treatment decisions.

## 2 Case description

In December 2022, a 52-year-old female patient was admitted to the Neurology Department of Chongqing University Affiliated Three Gorges Hospital, presenting with an acute “thunderclap” headache and vomiting. The patient had no history of smoking and maintained good overall health prior to admission.

**Figure 1:**
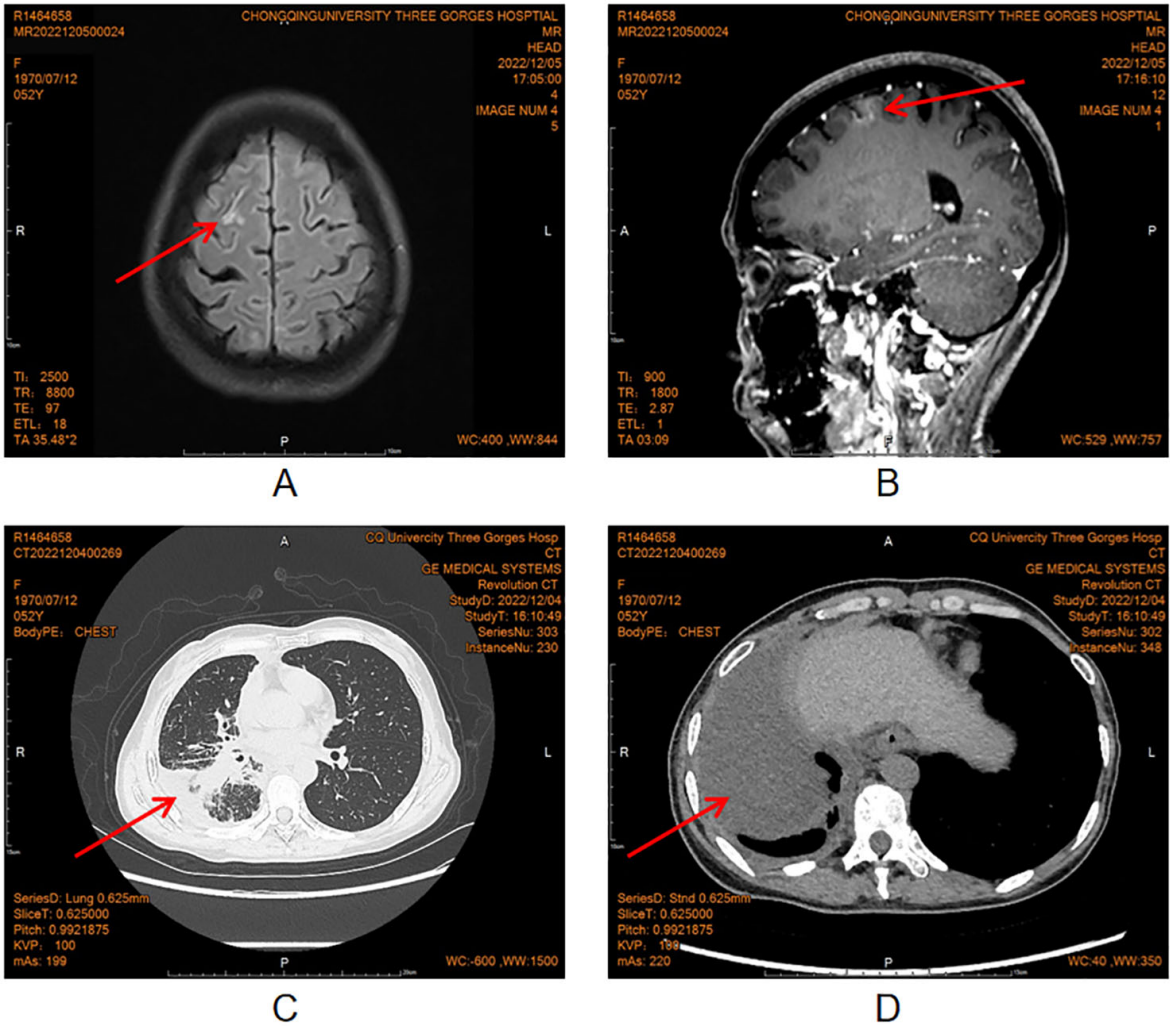
Head MRI and chest CT in December, 2022.

Upon presentation, her Eastern Cooperative Oncology Group Performance Status (ECOG PS) score was assessed at 4, with signs of elevated intracranial pressure. Following symptomatic management, a lumbar puncture was performed, revealing CSF pressure measured at 370 mmH2O and atypical cells within the CSF. Subsequent imaging studies revealed abnormal signals in the frontal, parietal, and occipital sulci of the cerebrum on head MRI, with widening noted in the right frontal lobe (**Figure 1**). Chest CT revealed increased scattered streaky density in the right lung with partial consolidation, suggestive of infectious lesions, and associated pleural effusion in the right thoracic cavity and interlobar fissure (**Figure 1**). Further positron emission tomography-computed tomography (PET-CT) scan showed increased tracer uptake of multiple tumor nodules localized in the right lower lobe (SUVmax 6.0) and extensive metastasis (SUVmax 7.3) (**Supplementary Figure 1**). In terms of pathological diagnosis, atypical cells were only detected in CSF (**Supplementary Figure 2**), but not in the right pleural effusion. Due to the patient’s poor condition, further tissue biopsy could not be performed, resulting in a lack of histopathological evidence for diagnosis. As the disease progresses, the patient experienced paroxysmal severe headaches, intermittent seizures accompanied by nausea and vomiting. Additionally, there was a gradual onset of blurred vision in the left eye and significant visual decline leading to blindness in the right eye.

Due to the patient’s critical condition and lack of sufficient pathological diagnosis, a multidisciplinary consultation (MDT) was conducted within the hospital. After MDT discussion, the patient was clinically diagnosed with a malignant tumor in the right lower lobe of the lung, which is highly likely to be NSCLC, and the tumor had spread to the right hilar lymph node, mediastinal lymph node, right pleura, multiple bones, and leptomeninges. Subsequently, the patient was transferred to the oncology department for further treatment. To enhance diagnostic accuracy and alleviate symptoms associated with elevated intracranial pressure, it was advised to implant an Ommaya reservoir for CSF drainage followed by subsequent ITC. On December 8, 2022, the patient underwent a ventriculostomy followed by the implantation of an Ommaya reservoir. The patient, a non-smoking Asian woman with lung cancer, had a higher probability of testing positive for the driver gene. Because of the critical condition, despite the unknown genetic mutation, she still bravely tried the third generation of epidermal growth factor receptor (EGFR) tyrosine kinase inhibitor (TKI). In light of evident LM symptoms requiring enhanced disease management measures; high-dose aumolertinib (165 mg/day) was initiated on December 29th 2022 as targeted therapy regimen. Fortunately after one week since treatment initiation, significant improvement was observed regarding “lightning strike” headache episodes along with alleviation in nausea/vomiting, and slight relief from blurred vision, and the ECOG PS dropped to 2 points, indicating that the general condition of the patient was significantly improved. Genetic testing results obtained on January 9, 2023 confirmed the presence of an EGFR-sensitive mutation in the patient. Specifically, EGFR exon 21 p.L858R was detected in both CSF and blood with mutation abundance of 41.15% and 0.78%, respectively.

February 10, 2023, CT examination showed pleural effusion were mostly absorbed, partial remission of lung lesions, complete remission of LM lesions. In addition, the intracranial pressure decreased significantly in retesting (370mmH2O before treatment vs. 220mmH2O after treatment). However, she performed an elevated carcinoembryonic antigen (CEA) level (707.8 ng/mL) in CSF. To further enhance the local control of LM, in the next two weeks, the patient received ITC with pemetrexed (30mg) via the Ommaya reservoir, 30mg on day 1 and day 8, with a cycle every 3 weeks. On February 21, 2023, the patient underwent a single cycle of systemic chemotherapy involving pemetrexed and carboplatin at dosages of 600mg and 300mg, respectively. Subsequently, we found that the CEA level in CSF dropped to 138.5 ng/mL. However, the patient developed severe myelosuppression (grade IV) with fever after chemotherapy, so systemic chemotherapy was discontinued in subsequent treatment, and targeted therapy combined with ITC by Ommaya reservoir was continued. Reexamination of imaging on April 19, 2023 showed a significant reduction in the lesions of pulmonary and intracranial tumors, but an expansion of bone metastasis and the emergence of significant lower back pain symptoms in the patient. In order to alleviate the lower back pain and reduce the risk of pathological fractures, the patient underwent palliative radiotherapy for some thoracolumbar vertebral metastases in May 2023.

**Figure 2:**
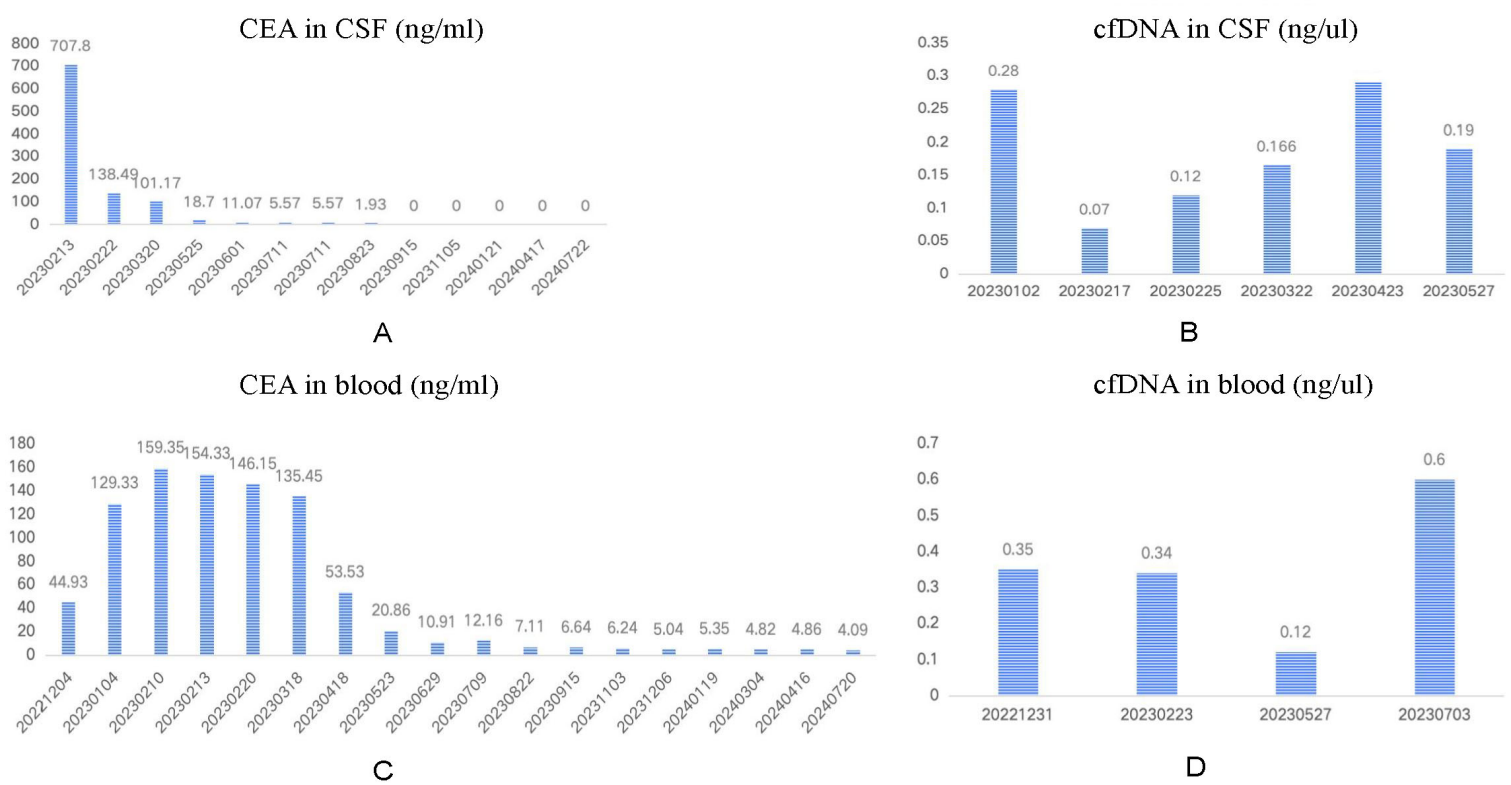
Dynamic monitoring of CEA and cfDNA in the CSF and in the blood.

Subsequently, considering the patient’s tolerance, the ITC of pemetrexed regimen via Ommaya reservoir was adjusted to 30 mg every 4 weeks starting July 2023, with the last chemotherapy on January 20, 2024. The patient continued oral high-dose aumolertinib (165 mg/day) during this period. Imaging showed improvement in lung and brain tumors while bone metastases remained stable; overall efficacy was evaluated as PR. Additionally, CEA levels in blood and CSF gradually normalized during follow-up (**Figure 2**), and no atypical cells were found in CSF after repeated examinations. cfDNA concentration in CSF significantly decreased early in treatment but slowly increased at 3 months before decreasing again at 5 months. In contrast, cfDNA levels in blood gradually decreased over the first 6 months of treatment followed by a rebound at month 7 (**Figure 2**).

**Figure 3:**
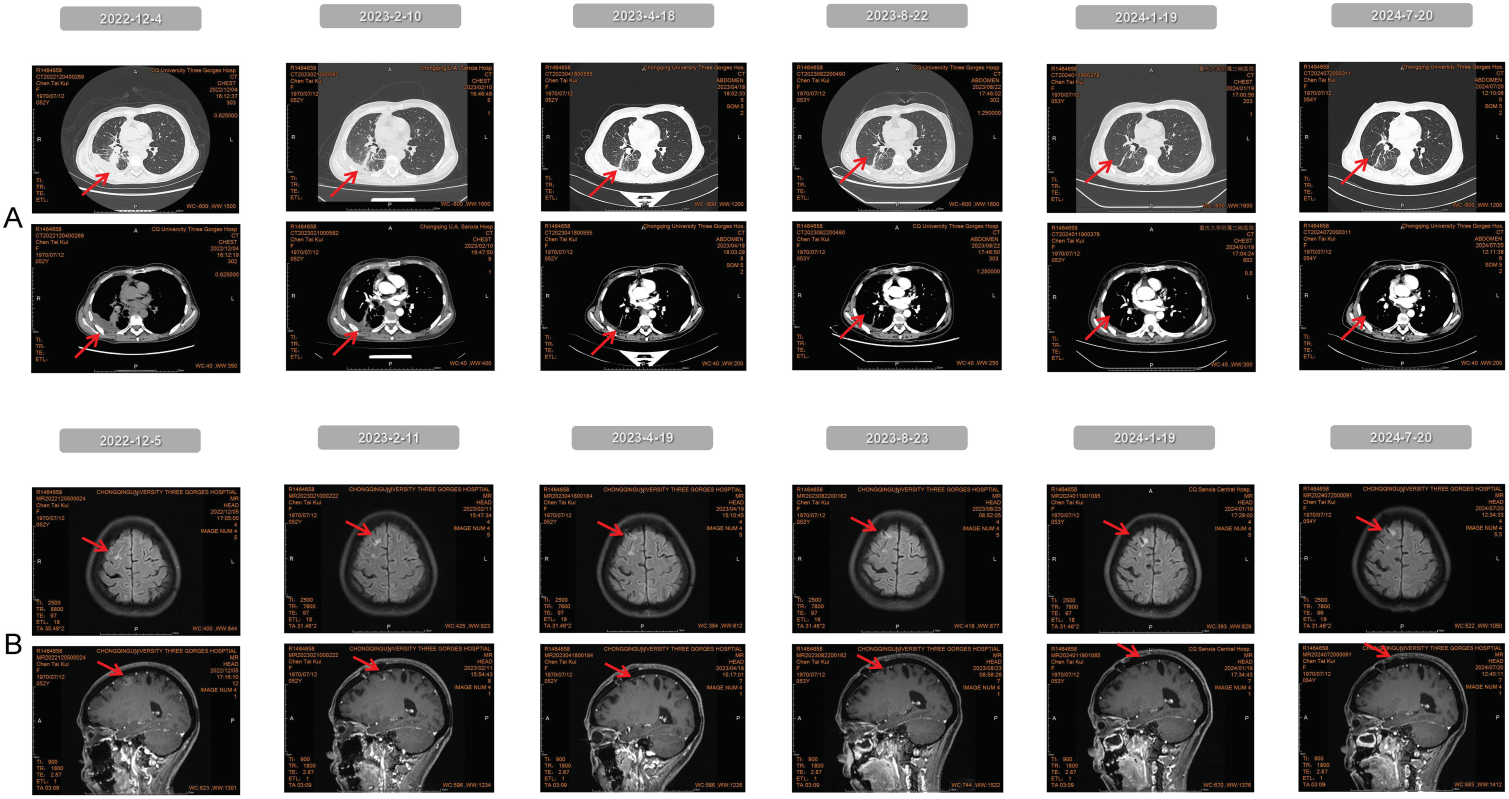
The imaging results obtained from the continuous follow-up.

During the single-dose ITC administered every 4 weeks, the patient’s adverse reactions resolved. The main treatment-related adverse reactions (TRAEs) were grade II myelosuppression and grade I gastrointestinal issues (nausea, vomiting, and oral ulcers), which improved with symptomatic treatment (specific TRAEs are listed in **Table 1**). The patient has an ECOG PS score of 1 and significant relief from headache, nausea, and vomiting. Her left eye vision notably improved while her right eye progressed from blindness to blurriness. Due to this positive response to treatment, she has been on a chemotherapy hiatus since February 2024 and is now receiving high-dose aumolertinib. The latest follow-up was on July 20, 2024. We have documented changes in clinical manifestations, imaging exams, and genetic testing via next-generation sequencing (NGS) throughout the treatment process (see **Table 2**, **Figure 3**, **Supplementary Table 1**).

**Table 1:** TRAEs.

Event	CTCAE 5.0	Possibility factor	Drug treatment/ self remission
WBC count decrease	4	Systemic chemotherapy, ITC or Aumolertinib	Drug treatment
Platelet count decrease	2	Systemic chemotherapy, ITC or Aumolertinib	Drug treatment
Anemia	2	Systemic chemotherapy, ITC or Aumolertinib	Drug treatment
Nausea	2	ITC	Drug treatment
Vomiting	2	ITC	Drug treatment
Oral ulcers	2	Aumolertinib or ITC	Drug treatment
Decreased appetite	2	Systemic chemotherapy, ITC or Aumolertinib	Drug treatment
Fatigue	2	Systemic chemotherapy, ITC or Aumolertinib	Self remission
ALT increase	1	Aumolertinib or ITC	Drug treatment
AST increase	1	Aumolertinib or ITC	Drug treatment

TRAEs, treatment-related adverse reactions; CTCAE 5.0, the common terminology criteria for adverse events version 5.0; ITC, intrathecal  chemotherapy.

**Table 2:** Records of important clinical manifestations of the patient.

Date	ECOG PS (0-4)	NRS of pain (0-10)	Nausea and vomiting (CTCAE 5.0) (1-5)	Pupil size (mm) and light reflex (sensitive +, slow -)	Eyesight
2022-12-09	4	6-7	4	Left: 3, +; Right: 6, -	Left: fuzzy; Right: blindness
2023-02-15	3	3-5	2	Left: 3, +; Right: 6, -	Left: light sense; Right: blindness
2023-04-16	2	1-2	1	Left: 3, +; Right: 5, -	Left: <4.0; Right: light sense
2023-06-20	1	1	1	Left: 3, +; Right: 5, -	Left: <4.0; Right: light sense
2023-09-25	1	0	1	Left: 3, +; Right: 4, -	Left: 4.0; Right: light sense
2023-12-30	1	0	1	Left: 3, +; Right: 4, -	Left: 4.0; Right: light sense
2024-03-28	1	0	0	Left: 3, +; Right: 4, -	Left: 4.0; Right: light sense
2024-06-25	1	0	0	Left: 3, +; Right: 4, -	Left: 4.0; Right: light sense
2024-07-20	1	0	0	Left: 3, +; Right: 4, -	Left: 4.0; Right: light sense

ECOG PS, eastern cooperative oncology group performance status; NRS, numerical rating scale; CTCAE 5.0, the common terminology criteria for adverse events version 5.0.

## 3 Discussion

LM is a devastating complication of NSCLC, with a low rate of early diagnosis and limited treatment options, resulting in poor prognosis. The incidence of LM in NSCLC without driver genes is 3.8%, while it can reach 9% in patients with EGFR mutations (3). Due to the low incidence rate, the rapid progress of the disease, and the heterogeneity of the LM population, there is currently no standard treatment protocol. In addition to symptomatic supportive therapy, active treatment strategies include systemic chemotherapy, ITC, whole brain radiation therapy (WBRT) and/or spinal axis radiation, as well as the EGFR-TKI for EGFR-positive patients. Radiotherapy is mainly used to alleviate symptoms from brain edema or focal lesion, however, there is limited evidence of its effectiveness in improving survival, and it increases the risk of toxicity (4–7). Some researchers suggest selecting suitable candidates for radiotherapy, noting that WBRT may benefit EGFR wild-type, nodular LM patients (8).

Systemic chemotherapy has limited efficacy for LM because it cannot achieve effective blood-brain barrier (BBB) penetration (1). However, ITC can bypass the BBB, achieving high CSF concentrations with low drug doses (9), enhancing local control of LM. Two main ITC methods exist: lumbar puncture and Ommaya reservoir. The Ommaya reservoir is more convenient and safer for CSF sampling and ITC. Emerging technologies like the Lumbar Intrathecal Port (LIP) are also becoming prominent in ITC (10). Due to dose-limiting toxicity and related complications, there is no consensus on the optimal dose, frequency, and duration of ITC. Classic ITC drugs include: thiotepa, methotrexate, (liposomal) cytarabine (3). Recently, pemetrexed has emerged as an important ITC agent. However, there is currently no consensus on the optimal dosage, administration frequency, and treatment duration. A phase I clinical study (11) showed that intrathecal injection of 10mg pemetrexed once or twice weekly resulted in favorable therapeutic effects and manageable toxicity in patients with LM. The response rate was 31% (4/13), with a disease control rate (DCR) of 54% (7/13). The most common AEs included bone marrow suppression, elevated liver transaminases, and neuritis. A single-arm phase 1/2 clinical trial (12) utilized ITC of 50 mg pemetrexed as the recommended dosage (RD). The regimen involved 50 mg on day 1 and 5 of the first week, followed by every three weeks for four cycles, then monthly until disease progression or intolerance. The mOS was 9.0 months. Primary AEs included nausea, vomiting, myelosuppression, and neurotoxicity (grade 1 or grade 2). Researchers have suggested administering 30 mg of pemetrexed on days 1 and 8 every three weeks, which has demonstrated positive effects in clinical practice (13). In this case, the patient initially received a 3-week pemetrexed regimen (30mg, D1, D8, q3W) for 4 cycles. In the maintenance stage, pemetrexed was adjusted to a 4-week schedule (30 mg, D1, D8, q4W) for 5 cycles. The 3-week regimen had a higher risk of myelosuppression, while the 4-week regimen was better tolerated. Therefore, prophylactic leukocyte-count support is crucial, especially for patients with multiple bone metastases. Folic acid and vitamin B12 supplementation should also be regularly administered to reduce AEs (11, 12).

For LM patients with EGFR-positive, third-generation TKIs have demonstrated superior efficacy compared to first- and second-generation TKIs. Small sample studies have reported LM-PFS of 2.0 to 2.3 months and mOS of 3.4 to 3.8 months for gefitinib, erlotinib, and afatinib (14–16). Third-generation EGFR-TKIs exhibit superior penetration through the BBB, making them more suitable for treating LM (3, 17). A retrospective study compared clinical outcomes of standard-dose osimertinib to first-generation TKIs in untreated EGFR-positive NSCLC with LM, showing superior mPFS (16.9 months vs. 8.6 months) and mOS (26.6 months vs. 20.0 months) in the osimertinib group (18). Researchers are attempting to improve disease management by increasing the concentration of EGFR TKI in CSF. A phase II study showed that osimertinib (160 mg/day) achieved an objective response rate (ORR) of 27.5% and a DCR of 82.5% in EGFR T790M-positive NSCLC with BM/LM, the mPFS was 5.7 months, and the common AEs included decreased appetite, diarrhea, and skin rash, most grade 1 or 2 levels (19). In a Phase I study (BLOOM) (6), osimertinib (160 mg/day) achieved an LM-ORR of 62% in EGFRm NSCLC patients with LM who had progressed on TKIs (n=41). The mPFS and mOS were 8.6 and 11.0 months. 24% of patients experienced ≥3 grade AEs, leading to discontinuation in 9 and dose reduction in 5 patients. A real-world study (n=48) showed that high-dose furmonertinib (240 mg/day) achieved an LM-ORR of 50.0%, an LM-DCR of 92.1%, and a mOS of 8.43 months in EGFR-mutated LM. 22 patients (45.8%) experienced TRAEs, and 3 (6.3%) had grade 3 AEs, leading to a dose reduction to 160 mg/day (20). Related studies indicate that aumolertinib is effective in controlling CNS metastasis, particularly at higher doses, although there is currently limited data on LM. In the AENEAS study (21), aumolertinib (110 mg/day) demonstrated significant improvement in CNS PFS compared to gefitinib when used as a first-line treatment for advanced NSCLC. The median CNS PFS was 29.0 months for aumolertinib and 8.3 months for gefitinib (HR=0.31; 95% CI, 0.17-0.56; P<0.001). The 12-month CNS PFS rates were 72.5% for aumolertinib and 30.4% for gefitinib. The ACHIEVE study (22) showed that high-dose aumolertinib (165 mg/day) administered as first-line treatment to patients with EGFR-positive NSCLC with BM resulted in a 12-month intracranial PFS (iPFS) rate of 75.0%, with the miPFS not reached. The ARTISTRY study (23) cohort 2 is currently enrolling 10 newly diagnosed patients with LM-NSCLC, with the initial treatment dose of aumolertinib set at 110mg/day. Patients will undergo efficacy assessments every 4 weeks until they progress, and if there are no PDs in two consecutive assessments, the dose can be gradually increased to 165/220mg/day ± radiation therapy. It is worth noting that,patients with the exon 19 mutation have a relatively better prognosis than those with the L858R mutation (4, 18). Based on these studies, third-generation EGFR-TKIs show superior efficacy and safety in patients with brain/meningeal metastases from EGFR-mutated NSCLC, and dose escalation may be a better treatment strategy. Further research is required to determine the optimal selection of TKIs, the best drug dosage, and the impact of different mutation types on TKIs efficacy in LM.

Monotherapy has a limited effect on improving the prognosis of LM, while a combination treatment model may provide greater benefit. Retrospective studies show that the use of ITC and EGFR-TKI are important predictors of good survival prognosis (7, 24). A retrospective analysis (9) showed that combining osimertinib (80mg/day) with ITC of methotrexate resulted in a mPFS of 10.8 months for LM patients who progressed after EGFR-TKI. This is comparable to the efficacy observed with 160 mg/day osimertinib in the BLOOM study, suggesting that combination therapy may offer better outcomes. Another retrospective analysis showed that in NSCLC patients who progressed to LM after osimertinib treatment (n=9), the combination of high-dose aumolertinib (220 mg/day) with ITC of pemetrexed, combined or not combined with bevacizumab resulted in a mPFS of 11 months and a mOS of 14 months (25). Additionally, relevant studies have shown that TKIs combined with anti-angiogenic drugs has better efficacy in LM. A retrospective real-world study (26) indicated that compared with EGFR-TKI alone, TKI combined with anti-angiogenic therapy (bevacizumab) experienced a delayed onset of LM (mOS1: 19.4 months vs. 13.9 months) and an extended survival after LM (mOS2: 14.5 months vs. 10.0 months). However, EGFR-TKI combined with systemic chemotherapy did not show a survival benefit advantage. A phase II prospective clinical trial (27) using osimertinib (80mg/day) plus bevacizumab (7.5mg/kg, q3W) for EGFRm NSCLC with LM (n=14) showed mLM-PFS of 9.3 months, LM-ORR of 50%, mOS of 12.6 months, and 1-year OS rate of 35.7%. Common AEs included leukopenia, thrombocytopenia, anemia, rash, anorexia, fatigue, ingrown toenail, hemoptysis/rhinorrhea, creatinine elevation, hypertension, and proteinuria. Grade 3 AEs were rare (one case of creatinine elevation, two cases of hypertension). Therefore, a combination of TKI with ITC or anti-angiogenesis therapy may be a more optimal treatment strategy for LM.

The diagnosis and prognosis of LM are crucial, but the unique nature of LM presents challenges for assessment. The Response Assessment in Neuro-Oncology (RANO) group has proposed a composite evaluation that includes three elements: standardized neurological examination, CSF cytology or flow cytometry, and radiographic evaluation (28, 29). However, the assessment criteria still lack clinical universality. Several studies show that tumor markers in CSF assist in diagnosing LM (1, 30, 31). In this case, a significant increase in CEA levels in CSF was noted at initial diagnosis. Moreover, the detection of cfDNA in CSF was crucial since the patient could not provide pathological tissue at that time. Studies indicate that liquid biopsy technology can achieve sensitivity as high as 93% (32). In our patient group, EGFR exon 21 mutations were detected in the CSF, with a significantly higher mutation load than in the blood. During treatment, two co-mutations were detected in CSF but not in blood. A small prospective study (n=21) indicated that the detection rate of EGFR mutations in CSF circulating tumor DNA (ctDNA) was about 2.4 times higher than that in blood (33). CSF testing more accurately reflects molecular changes associated with meningeal metastasis and offers better prognostic value than blood tests. For a thorough evaluation of patients with LM, combined CSF and blood tests are recommended. A significant correlation exists between tumor cell clearance in CSF and dynamic changes in CEA levels, which are key indicators for monitoring treatment efficacy and prognosis. While dynamic changes in cfDNA concentration in CSF were not significantly linked, studies suggest its potential prognostic value (34). Furthermore, circulating tumor cells (CTCs) and pro-inflammatory cytokines in CSF also correlated with LM patient prognosis (34, 35). Thus, alongside CSF cytology and tumor markers, csfDNA testing can be an essential diagnostic and management tool for LM.

Patients with NSCLC and LM exhibit significant heterogeneity, leading to varied clinical outcomes. In this case, the patient was diagnosed with LM initially, indicating high malignancy and aggressive tumor behavior that complicate treatment. However, after targeted therapy and ITC, the patient experienced prolonged PFS. A retrospective Korean study found that NSCLC patients with initial LM do not always have a poor prognosis; some achieved OS exceeding 12 months (36). Future research should further investigate the clinical and molecular characteristics of this subgroup. Patients with targetable mutations presenting with initial LM may be suitable candidates for aggressive therapeutic interventions.

In summary, LM is a hidden and dangerous disease, making early identification and intervention essential. Treatment presents both challenges and opportunities, and molecularly stratified treatment is recommended for NSCLC patients. We are currently conducting a prospective, open-label clinical trial titled “Aumolertinib Combined Intrathecal Chemotherapy for Leptomeningeal Metastasis From EGFR-Mutated NSCLC and Prognostic Value of Dynamic Changes in cfDNA Profiles “ (NCT05810350). Preliminary results have been presented at the 2023 and 2024 WCLC, and look forward to the future results. This case is one of our trial participants. Our findings suggest that combining high-dose amorafenib with intrathecal chemotherapy via the Ommaya reservoir may be a promising treatment for EGFR-positive NSCLC patients with LM. Monitoring CSF cfDNA using NGS technology aids in the diagnosis and treatment of LM patients. More randomized controlled trials are needed to further explore TKI drug selection, dosage, combination strategies, and achieve an appropriate balance between efficacy, quality of life, and cost-effectiveness.

## Data Availability

The original contributions presented in the study are included in the article/**Supplementary Material**. Further inquiries can be directed to the corresponding authors.
