# Correction: Case report: A case report and literature review on the efficacy of high-dose aumolertinib combined intrathecal pemetrexed by Ommaya reservoir for EGFR-mutated NSCLC with leptomeningeal metastasis as the initial symptoms

**DOI:** 10.3389/fonc.2025.1720422

**Published:** 2025-11-07

**Authors:** Maoxi Zhong, Li Zhou, Jing Guo, Chuan Chen, Yi Liu, Xiaoping Huang, Wei Wang

**Affiliations:** 1 Department of Cancer Center, Chongqing University Three Gorges Hospital, Chongqing, China; 2 School of Medicine, Chongqing University, Chongqing, China; 3 Chongqing Municipality Clinical Research Center for Geriatric Diseases, Chongqing, China; 4 Department of Thoracic Surgery, Chongqing University Three Gorges Hospital, Chongqing, China

**Keywords:** high-dose aumolertinib, intrathecal chemotherapy, leptomeningeal metastasis, NSCLC, case report

In the published article, there was an error in the author list, and author order was erroneously given as:

“Maoxi Zhong^1,2,3^, Li Zhou^1,2,3^, Jing Guo^1,2,3^, Chuan Chen^1,2,3^, Wei Wang^1,2,3*^, Xiaoping Huang^1,2,3*^ and Yi Liu^2,3,4*”^

The correct author list reads:

The corrected author list appears below.

“Maoxi Zhong^1,2,3^, Li Zhou^1,2,3^, Jing Guo^1,2,3^, Chuan Chen^1,2,3^, Yi Liu^2,3,4*^, Xiaoping Huang^1,2,3*^ and Wei Wang^1,2,3*”^

The authors apologize for this error and state that this does not change the scientific conclusions of the article in any way. The original article has been updated.

