# Supplementary figures and images for: Case report: A case report and literature review on the efficacy of high-dose aumolertinib combined intrathecal pemetrexed by Ommaya reservoir for EGFR-mutated NSCLC with leptomeningeal metastasis as the initial symptoms
Source: Front Oncol. 2025 Jan 30;15:1502934. doi: 10.3389/fonc.2025.1502934 (PMC11821504; doi:10.3389/fonc.2025.1502934)

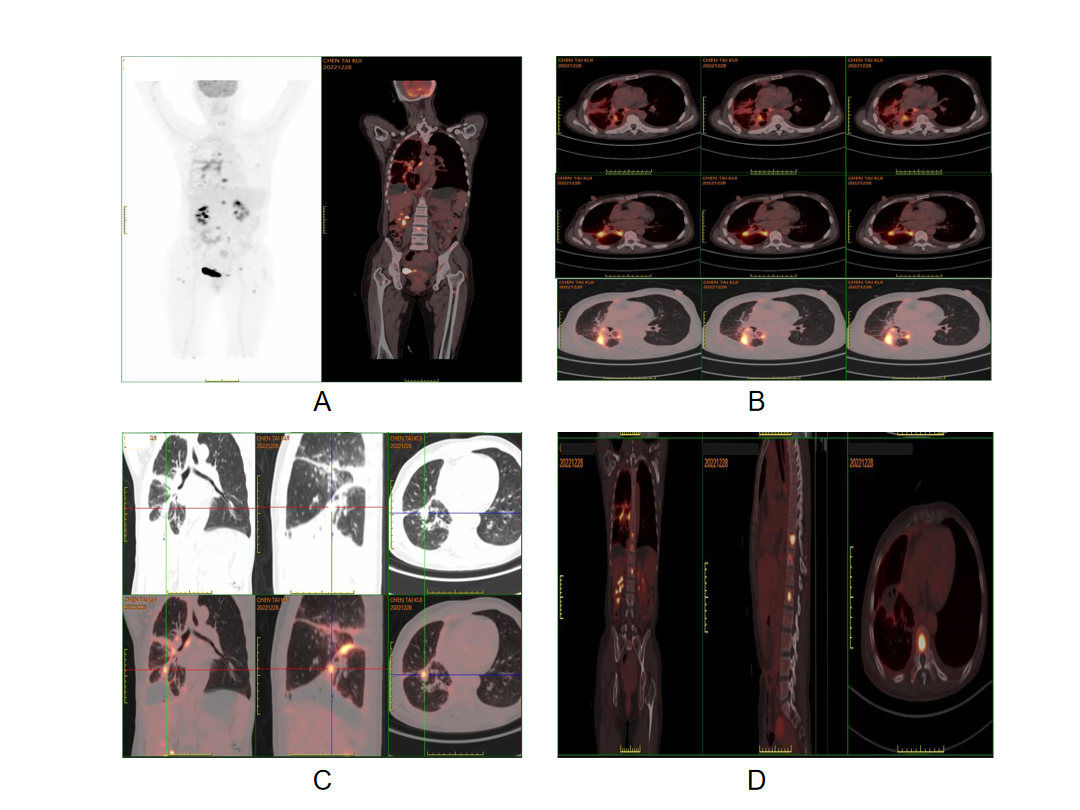

Supplement: Supplementary file 1 [file Image1.tif]

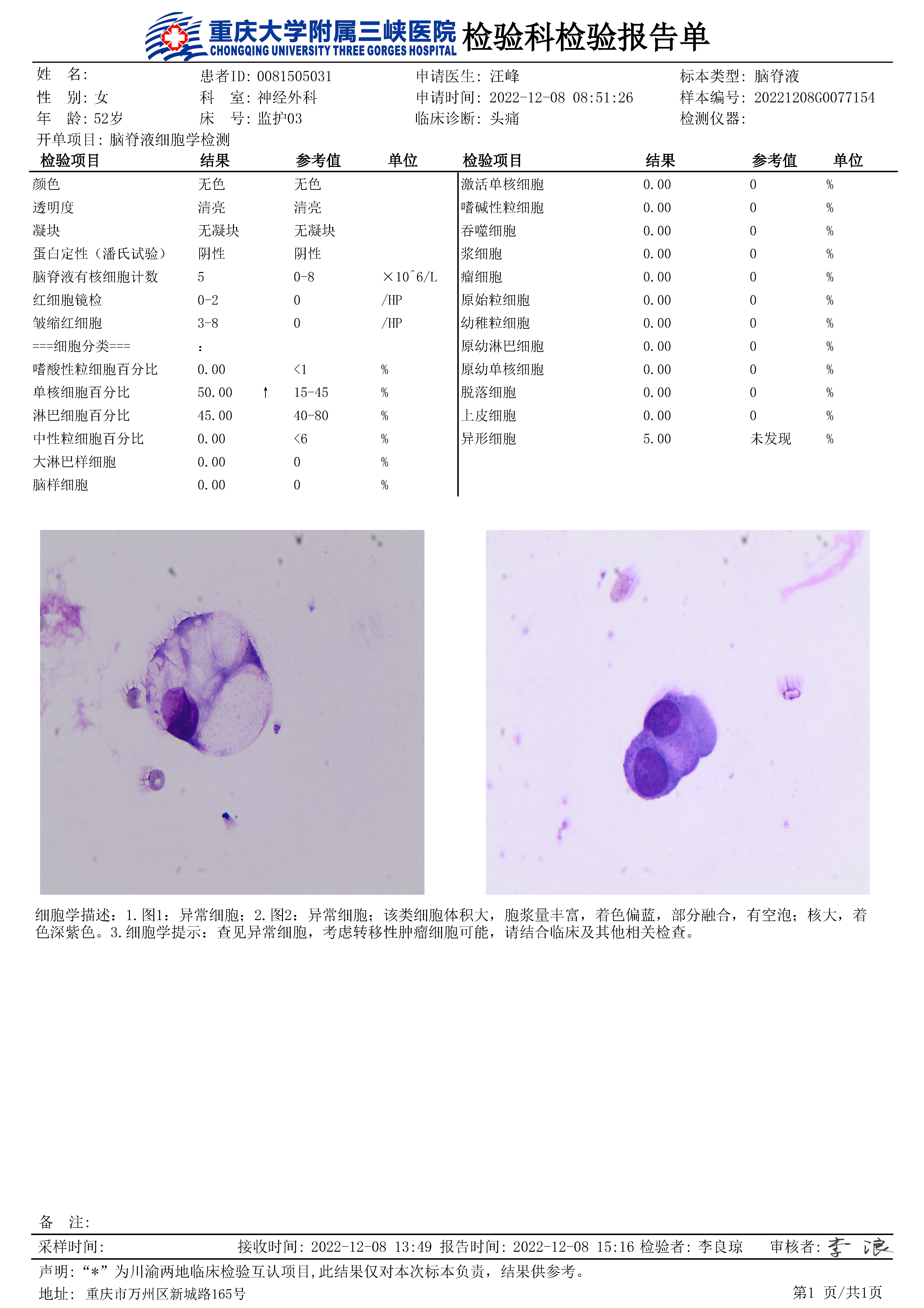

Supplement: Supplementary file 2 [file Image2.tiff]
