# Supplementary material for: Case report: A case report and literature review on the efficacy of high-dose aumolertinib combined intrathecal pemetrexed by Ommaya reservoir for EGFR-mutated NSCLC with leptomeningeal metastasis as the initial symptoms
Source: Front Oncol. 2025 Jan 30;15:1502934. doi: 10.3389/fonc.2025.1502934 (PMC11821504; doi:10.3389/fonc.2025.1502934)
Supplement: Supplementary file 3 [file Table1.docx]

Supplementary Table 1. Dynamic monitoring and detection results of tumor driving genes.

| **Gene, exon, base, amino acid** | **EGFR**  **exon21 c.2573T>G p.L858R** | **TP53**  **exon9**  **c.951 952 delGCinsA p.P318fs** | **TP53**  **exon9**  **c.951 delG p.Q317fs** | **DNMT 3A exon23 c.2645G>A p.R883H** |
| --- | --- | --- | --- | --- |
| Blood （2022-12-31） | 0.78% | - | - | - |
| CSF （2023-01-02） | 41.15% | 92.62% | - | - |
| CSF （2023-02-17） | 40.33% | 87.68% | - | - |
| Blood （2023-02-23） | - | - | - | - |
| CSF （2023-02-25） | 39.33% | 70.51% | - | - |
| CSF （2023-03-22） | 41.76% | - | 84.00% | - |
| CSF （2023-04-23） | 26.12% | 51.55% | - | - |
| Blood （2023-05-27） | - | - | - | - |
| CSF （2023-05-27） | - | - | - | 1.37% |
| Blood （2023-07-03） | - | - | - | - |
